# A mitochondrial checkpoint to NF-κB signaling

**DOI:** 10.1038/s41419-024-06868-3

**Published:** 2024-07-03

**Authors:** Emma Guilbaud, Lorenzo Galluzzi

**Affiliations:** 1 Department of Radiation Oncology, Weill Cornell Medicine, New York, NY USA; 2 Sandra and Edward Meyer Cancer Center, New York, NY USA; 3 Caryl and Israel Englander Institute for Precision Medicine, New York, NY USA

**Keywords:** Cancer microenvironment, Tumour immunology, Apoptosis

## Abstract

Mitochondrial dysfunction can elicit multiple inflammatory pathways, especially when apoptotic caspases are inhibited. Such an inflammatory program is negatively regulated by the autophagic disposal of permeabilized mitochondria. Recent data demonstrate that the ubiquitination of mitochondrial proteins is essential for NEMO-driven NF-kB activation downstream of mitochondrial permeabilization.

Mitochondrial outer membrane permeabilization (MOMP) is a key regulator of intrinsic apoptosis. MOMP occurs upon the oligomerization of BCL2 associated X, apoptosis regulator (BAX) and BCL2 antagonist/killer 1 (BAK1) on the outer mitochondrial membrane (OMM), a process that is inhibited by BCL2 apoptosis regulator (BCL2), BCL2 like 1 (BCL2L1, best known as BCL-X_L_) and MCL1 apoptosis regulator, BCL2 family member (MCL1) [1]. Besides precipitating apoptotic cell death, MOMP promotes the cytosolic accumulation of multiple pro-inflammatory molecules, including (but not limited to) mitochondrial DNA and RNA species, as well as cardiolipin [2, 3]. Importantly, several systems are in place to prevent MOMP from eliciting unwarranted inflammatory reactions in physiological scenarios. On the one hand, cells adapting to sublethal stress conditions, in which MOMP involves a limited fraction of the mitochondrial network (so-called “minority MOMP”) [4], can rapidly degrade permeabilized mitochondria (and hence limit the availability of pro-inflammatory molecules) through an ubiquitin-dependent specialized variant of autophagy commonly known as mitophagy [5, 6]. On the other hand, cells succumbing to MOMP during physiological waves of apoptosis, for instance as underlying the renewal of epithelial layers, massively activate post-mitochondrial caspases, notably caspase 3 (CASP3), which has multipronged anti-inflammatory effects [3, 7].

One of the central mechanism through which MOMP elicits inflammation (in the context of caspase inhibition) involves the activation of an NF-κB-dependent transcriptional program elicited by inhibitor of nuclear factor kappa B kinase subunit beta (IKBKB, best known as IKKβ) and inhibitor of nuclear factor kappa B kinase regulatory subunit gamma (IKBKG, best known as NEMO) that culminates with the secretion of tumor necrosis factor (TNF) [8, 9]. Recent data from Vringer and colleagues demonstrate that the NEMO-driven activation of NF-κB elicited by MOMP in caspase-deficient settings relies on the ubiquitination of various OMM proteins as a prelude to mitochondrial degradation [10].

To investigate the precise mechanisms through which MOMP elicits mitochondrial disposal, Vringer and collaborators harnessed human osteosarcoma U2OS cells optionally subjected to the co-deletion of *BAX* and *BAK1* and exposed them to ABT-737 and S63845 (which together inhibit most anti-apoptotic BCL2 family members) plus the pan-caspase inhibitor QVD. Immunoblotting, PCR and immunofluorescence microscopy confirmed that (at least in caspase-defective settings) MOMP elicits mitochondrial degradation in a BAX- and BAK1-dependent manner, a process that is accompanied by extensive ubiquitination of mitochondrial proteins. Intriguingly, the loss of mitochondrial protein as elicited by ABT-737/S63845/QVD in U2OS cells could not be rescued by the deletion of *ATG5* or *ATG7* (which encode two key components of the autophagic machinery), but was at least in part sensitive to proteasomal inhibition, suggesting a limited involvement of canonical mitophagy in this specific experimental setting. Of note, similar results have recently been obtained in human cervical carcinoma HeLa cells treated with ABT-737 plus QVD [11]. Proteomic assessments based on a mouse immortalized endothelial cell line (SVEC4-10 cells) demonstrated that >75% proteins that were ubiquitinated upon ABT-737/S63845/QVD exposure localized to mitochondria, while most other cellular compartments appeared to rather undergo deubiquitination [10].

Next, Vringer and co-authors harnessed immunoblotting and immunofluorescence microscopy to obtain further insights into the specific type of ubiquitin linkage elicited at mitochondria experiencing MOMP in caspase-deficient settings, identifying K63-linked and (to a lower extent) M1-linked ubiquitin as the major species. Importantly, mitochondrial ubiquitination as elicited by ABT-737/S63845/QVD in U2OS cells was accompanied by the BAX- and BAK1-dependent recruitment of NEMO at mitochondria, as demonstrated by fluorescence microscopy after expression of a GFP-tagged NEMO variant and by immunoblotting upon subcellular fractionation. In both U2OS and SVEC4-10 cells, this process and the consequent activation of NF-κB, as assessed via the nuclear relocalization of RELA proto-oncogene, NF-kB subunit (RELA, best known as p65), relied on both NEMO domains involved in K63 ubiquitin binding. In line with this notion, pharmacological inhibition of E1 (which catalyzes the first step in K63 ubiquitination) prevented NEMO mitochondrial translocation driven by ABT-737/S63845/QVD, but the same did not hold true when the gene encoding ring finger protein 31 (RNF31, best known as HOIP), which is required for M1 ubiquitination, was deleted in mouse embryonic fibroblasts [10]. These findings suggest that K63 (but not M1) ubiquitin linkages are required for NEMO-dependent signaling driven by MOMP in caspase-defective scenarios.

That said, a large panel of SVEC4-10 clones lacking established mitochondrial E3 ligases, including mitochondrial E3 ubiquitin protein ligase 1 (MUL1), membrane associated ring-CH-type finger 5 (MARCH5), and X-linked inhibitor of apoptosis (XIAP), failed to enable the identification of the E3 ligase involved in this process. Similarly, SVEC4-10 cells lacking PTEN induced kinase 1 (PINK1), which promotes ubiquitination at the OMM via ubiquitin phosphorylation and activation of parkin RBR E3 ubiquitin protein ligase (PRKN), or mitogen-activated protein kinase kinase kinase 14 (MAPK3K14), which drive non-canonical NF-κB signaling upon MOMP, did not show defects in MOMP-driven mitochondrial NEMO translocation [10]. Thus, the identity of the E3 ligase underlying OMM protein ubiquitination as elicited by MOMP in the context of caspase inhibition remains unclear.

Finally, Vringer and colleagues investigated the precise mechanisms through which MOMP, which is associated with numerous bioenergetic and ionic consequences for mitochondria, elicits OMM protein ubiquitination and NEMO translocation. SVEC4-10 cells exposed to inhibitors of the mitochondrial respiratory chain or activators of mitochondrial Ca^2+^ uptake did not exhibit NEMO translocation. Moreover, the mitochondrial recruitment of NEMO elicited by ABT-737/S63845/QVD was insensitive to the mitochondrial permeability transition (MPT) inhibitor cyclosporin A. Conversely, SVEC4-10 cells responding to raptinal, which elicits MOMP in a BAX- and BAK1-independent manner, plus QVD exhibited robust NEMO recruitment and NF-κB activation irrespective of BAX and BAK1 expression [10]. Thus, mitochondrial integrity is the main regulator of OMM protein ubiquitination and consequent NEMO-dependent NF-κB signaling, at least in caspase-defective conditions.

Altogether, the findings from Vringer and colleagues define a novel mechanism through which – prior to degradation – the OMM of permeabilized mitochondria undergoes extensive ubiquitination in support of pro-inflammatory NF-κB activation, a process that depends on BAX and BAK1 and is tonically inhibited by caspases (Fig. 1). Of note, previous data suggest that NF-κB signaling can promote the mitophagic disposal of mitochondria experience MOMP by promoting the expression of the autophagic adaptor and ubiquitin-binding protein sequestosome 1 (SQSTM1, best known as p62), as part of a negative feedback loop to suppress MOMP-driven inflammasome activation [12]. In this context, while ATG5 and ATG7 appeared to be dispensable for the degradation of mitochondrial proteins as elicited by ABT-737/S63845/QVD in U2OS cells, it would be interesting to see whether mitophagy activation as elicited by urolithin A would quench NF-κB signaling as a consequence of restored mitochondrial degradation. Irrespective of this and other open questions, it appears that the so-called mitochondrial checkpoint is fundamental for the preservation of inflammatory homeostasis in a variety of pathophysiological settings.

**Fig. 1:**
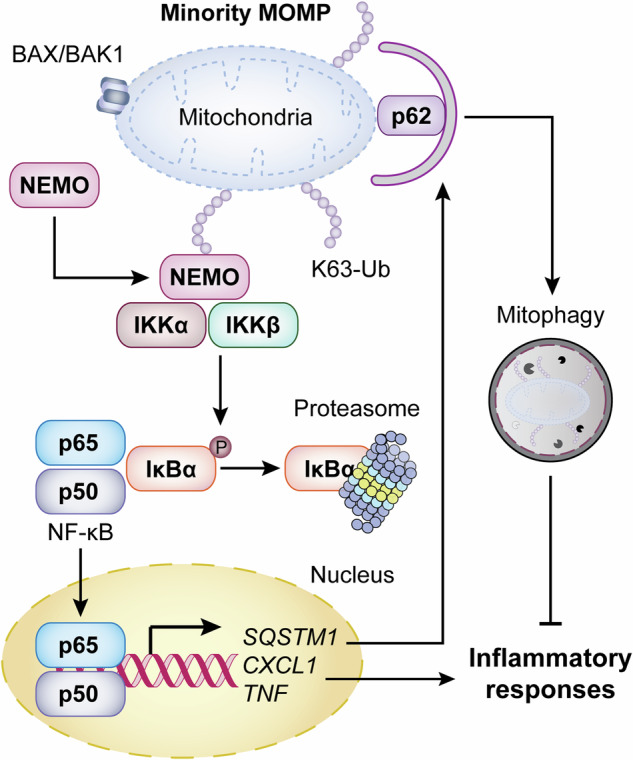
NF-κB signaling and the mitochondrial checkpoint. In caspase-incompetent scenarios, the loss of mitochondrial integrity can elicit various inflammatory effects including (but not limited to) NF-κB activation. Such a process does not rely on alterations of mitochondrial bioenergetics or calcium buffering, but rather involves the K63-linked ubiquitination (K63-Ub) of various outer mitochondrial membrane (OMM) proteins and the consequent recruitment of nuclear factor kappa B kinase regulatory subunit gamma (IKBKG, best known as NEMO). At least in some cells, this results in the activation of a negative feedback loop promoting the mitophagic disposal of permeabilized mitochondria via NF-κB-dependent sequestosome 1 (SQSTM1, best known as p62) expression. Thus, mitochondrial integrity stands at a central position in the preservation of inflammatory homeostasis. BAK1, BCL2 antagonist/killer 1 (BAK1); BAX, BCL2 associated X, apoptosis regulator; CXCL1, C-X-C motif chemokine ligand 1; IKBα (official name: NFKB inhibitor alpha), NFKB inhibitor alpha; IKKα (official name: CHUK), component of inhibitor of nuclear factor kappa B kinase complex; IKKβ (official name: IKBKB), inhibitor of nuclear factor kappa B kinase subunit beta; P, inorganic phosphate; p50 (official name: NFKB1), nuclear factor kappa B subunit 1; p65 (official name: RELA), RELA proto-oncogene, NF-kB subunit; TNF, tumor necrosis factor.
